# Mark-Release-Recapture Reveals Extensive Movement of Bed Bugs (*Cimex lectularius* L.) within and between Apartments

**DOI:** 10.1371/journal.pone.0136462

**Published:** 2015-09-09

**Authors:** Richard Cooper, Changlu Wang, Narinderpal Singh

**Affiliations:** Department of Entomology, Rutgers University, New Brunswick, New Jersey, United States of America; University of Tours, FRANCE

## Abstract

Understanding movement and dispersal of the common bed bug (*Cimex lectularius* L.) under field conditions is important in the control of infestations and for managing the spread of bed bugs to new locations. We investigated bed bug movement within and between apartments using mark-release-recapture (m-r-r) technique combined with apartment-wide monitoring using pitfall-style interceptors. Bed bugs were collected, marked, and released in six apartments. The distribution of marked and unmarked bed bugs in these apartments and their 24 neighboring units were monitored over 32 days. Extensive movement of marked bed bugs within and between apartments occurred regardless of the number of bed bugs released or presence/absence of a host. Comparison of marked and unmarked bed bug distributions confirms that the extensive bed bug activity observed was not an artifact of the m-r-r technique used. Marked bed bugs were recovered in apartments neighboring five of six m-r-r apartments. Their dispersal rates at 14 or 15 d were 0.0–5.0%. The estimated number of bed bugs per apartment in the six m-r-r apartments was 2,433–14,291 at 4–7 d after release. Longevity of bed bugs in the absence of a host was recorded in a vacant apartment. Marked large nymphs (3^rd^– 5^th^ instar), adult females, and adult males continued to be recovered up to 57, 113, and 134 d after host absence, respectively. Among the naturally existing unmarked bed bugs, unfed small nymphs (1^st^– 2^nd^ instar) were recovered up to 134 d; large nymphs and adults were still found at 155 d when the study ended. Our findings provide important insight into the behavioral ecology of bed bugs in infested apartments and have significant implications in regards to eradication programs and managing the spread of bed bugs within multi-occupancy dwellings.

## Introduction

The behavioral ecology of the bed bug (*Cimex lectularius* L.) in naturally infested dwellings is poorly understood. Much of what we do know dates back to research conducted nearly 50 years ago or more [1–7]. While there has been an increase in research as a result of the recent resurgence of bed bugs, research on their activity in naturally infested dwellings has been limited [8].

Wang et al. [9] used both visual inspection and an experimental pitfall-style trap placed under the legs of beds and furniture to quantify changes in the number of bed bugs in 16 infested apartments. Ninety-nine percent of the bed bugs found during visual inspection were associated with beds and upholstered furniture, however the pitfall trap catch suggested that a higher percentage of bed bugs were coming from areas off the bed than from on the bed. More recently, pitfall-style interceptor traps were shown to be effective at trapping bed bugs when placed in locations away from the beds and upholstered furniture [10–14]. These studies suggest that bed bugs can travel extensively within infested apartments, and that passive pitfall-style traps can be used to monitor bed bug movement both toward and away from host sleeping and resting areas.

Haynes et al. [15] reported that under laboratory conditions bed bugs can travel up to 4.9 m in five minutes, and suggested that they can travel greater distances during the night, when they are active for hours at a time. Pfiester et al. [16] investigated aggregation behavior under laboratory conditions and suggested that first instar nymphs were the least likely developmental stage to disperse, and that recently fed adult females were the most likely to move away from aggregations. In another laboratory study, How and Lee [17] examined distance traveled by various stages of the tropical bed bug (*Cimex hemipterus* (F.)), a species closely related to the common bed bug. They concluded that distance traveled varied significantly based upon the developmental stage, adult sex, and feeding status. Similar to Pfiester et al. [16], How and Lee [17] concluded that early instars were the least likely to disperse and recently fed females were the most likely to disperse.

A limited number of studies have examined bed bug dispersal behavior under field conditions [3], [18–19]. Naylor [18] provided evidence that both nymphs and adults of both sexes disperse based upon bed bugs captured on sticky traps located in a common hallway of an apartment building outside of two infested apartments. Lehnert [19] found 18 lone bed bugs away from aggregations in eight infested apartments but did not report the distance between the nearest aggregation and the location of the lone bugs. Potter et al. [12] provided some insight into the bed bug movement within apartments by marking bed bugs with different colored paints, and then using pitfall traps and visual inspection to detect bed bug movement in two heavily infested residences. During the one week study, they found bed bugs that had moved up to 9.1 m away from their original resting location.

Active dispersal of bed bugs from infested apartments to neighboring apartments has been implicated as one of the causes for the spread of bed bugs within communities. The spread of bed bugs from one unit to 68 units was tracked in a medical school housing facility over a 25 mo period [20]. Similarly, bed bugs spread from a single apartment to 53% of the apartments in a 223 unit building in just 41 mo [14]. In both studies, over 50% of the infested living units shared a common wall, floor or ceiling with another infested apartment [14], [20]. The first evidence that active dispersal may be in part responsible for widespread infestations was provided by Wang et al. [14] who captured bed bugs in interceptor traps located behind the entry door inside infested apartments, as well as in traps located in the hallway just outside of infested apartments. Naylor [18] also believed that bed bugs captured on sticky traps located in a common hallway of an apartment building had dispersed from a heavily infested apartment. Molecular studies have provided additional evidence in support of active dispersal of bed bugs between apartments [21–22]. Collection of bed bugs from infestations in different apartments within the same apartment building expressed low genetic diversity, suggesting that widespread infestations within apartment buildings were most likely to have resulted from a single introduction [21]. In another study using microsatellite DNA markers to screen bed bug populations in apartment buildings with widespread infestations, it was suggested that bed bugs were actively dispersing between neighboring apartments above, below, adjacent, or within a short distance of an infested unit [22]. In spite of the existing evidence for active dispersal, absolute proof is still lacking.

In this study, we first evaluated the ability of interceptors in catching different bed bug developmental stages and the effect of marking procedure on mortality of bed bugs. We then used m-r-r technique in both vacant and occupied apartments to study the movement of bed bugs within and between apartments, to estimate population size, and to examine the longevity of bed bugs in the absence of a host.

## Materials and Methods

### Ethics Statement

The field study received Rutgers University IRB approval (protocol # E11-766). Permission to perform the m-r-r study was granted from the housing authorities that participated in the study. Consent was obtained from residents whose apartments were used in the study and they were compensated either US $50 or $200 in appreciation for their time and cooperation.

### Laboratory Bioassays

#### 1. Reliability of interceptors for estimating bed bug population structure

Climbup interceptors (Susan McKnight, Inc., Memphis, TN, USA) referred to hereafter as interceptors, were used throughout this study to sample bed bugs in apartments. We carried out two laboratory assays to examine: 1) if different stages and sexes are equally trapped by interceptors, and 2) determine the escape capability of bed bugs that had fallen into interceptors.

A strain of bed bugs collected between 2008 and 2010 from apartments in Indianapolis, IN, was used in the test. They were maintained in round plastic containers (4.7 × 5 cm) (Consolidated Plastics, Stow, OH, USA) with folded construction paper (Universal Stationers Supply Co., Deerfield, IL, USA) as harborages at 26 ± 1°C, 40 ± 10% RH, and a 12:12 h (L:D) photoperiod. They were fed weekly on defibrinated rabbit blood (Hemostat Laboratories, Dixon, CA, USA) using a Hemotek membrane-feeding system (Discovery Workshops, Accrington, UK). The bed bugs were starved for six days prior to the experiment, as starved bed bugs are less likely to aggregate [23].

1.1 Interceptor trap catch. The study was conducted in a non-ventilated room at 24–25°C and a 12:12 h (L:D) photoperiod. Each day carbon dioxide (CO_2_) at the rate of 100 ml/min was released in the center of the room during the dark cycle using methods described in Singh et al. [24] to stimulate bed bug foraging activity [24], [25].

The bioassay was conducted in plastic arenas (80 × 75 × 5 cm) with bottoms lined with brown paper and a layer of fluoropolymer resin (BioQuip products, Rancho Dominguez, CA, USA) applied to inner walls to prevent bed bugs from escaping. Four interceptors were placed in each arena, with one in each corner. Aged interceptors were used to mimic the condition that interceptors would be in under field conditions. Interceptors were aged prior to the experiment, by placing them in occupied apartments for two weeks and then retrieving them for the experiment. One hundred and fifty bed bugs (40 1^st^ instars, 40 3^rd^-5^th^ instars, 35 adult males, and 35 adult females) were contained in the center of the arena with a plastic ring (6.4 × 13.3 cm) at one hour into the dark cycle. After one hour conditioning period, the plastic ring was removed. Six arenas were used to provide six replications.

After four days, the number of bed bugs trapped in interceptors and those remaining in each of the arenas were counted by developmental stage and adult sex. Bed bugs not captured were removed from the arenas, while those captured in interceptors were left in place to examine escape rates of captured bed bugs in the next experiment.

1.2 Escape of bed bugs from interceptors. The six arenas containing interceptors with trapped bed bugs from the previous experiment were moved to a ventilated room at 23–25°C, 40% RH, and 12:12 h (L:D) photoperiod. During the experiment, CO_2_ was released in the same manner as the previous experiment. Eight folded paper harborages (5.1 × 3.3 cm) were placed along the edges of the arena floor (two per side) and another in the center of the arena. The paper harborages were conditioned with bed bug feces and were used in the experiment to stimulate movement and subsequent arrestment [26] of bed bugs escaping from interceptors. Prior to the being used in the assay the harborages had been used in rearing containers for immature bed bugs, thus each harborage contained numerous bed bug feces but no eggs. The number of live and dead bed bugs, in and outside of interceptors and on paper harborages, was recorded by developmental stage and adult sex at 24 h and then every two days for the next ten days. Bed bugs that were unable to crawl when gently prodded were considered dead. After each observation, bed bugs that escaped from interceptors were removed from the arenas.

#### 2. Effect of marking procedure on bed bug survival

**Fig 1:**
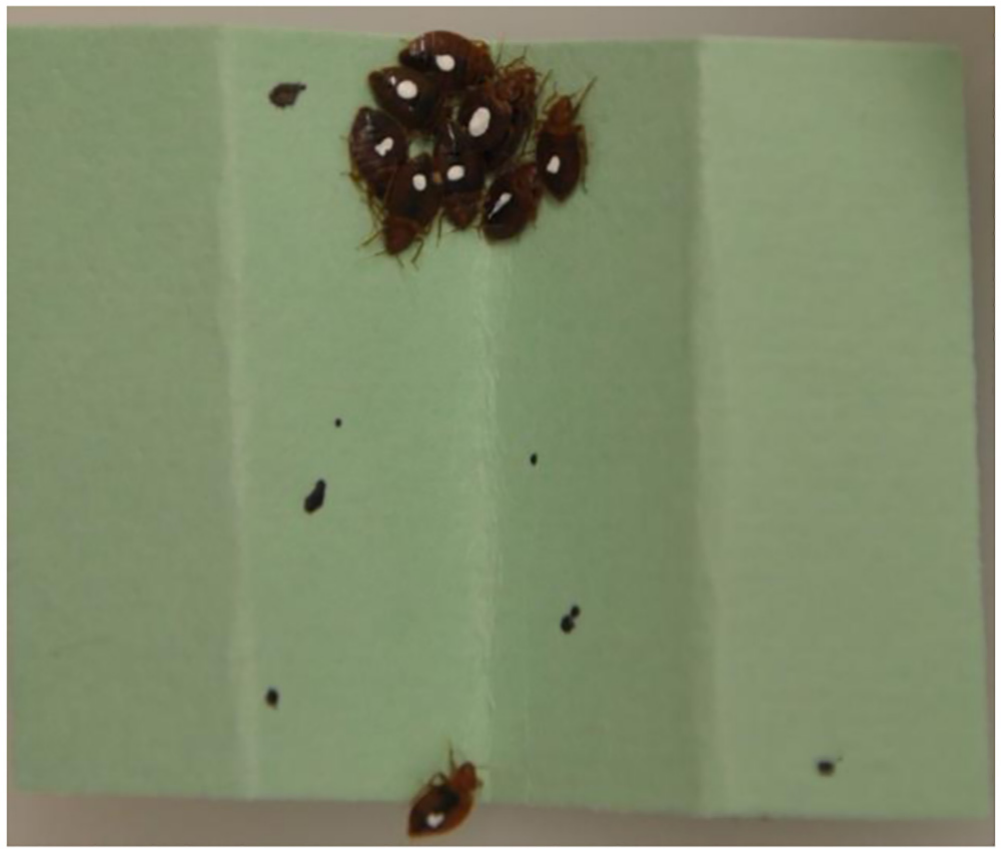
Marked bed bugs.

The excess bed bugs collected from one of the m-r-r apartments were divided into two groups: a marked group (22 males, 19 females) and an unmarked group (24 males, 25 females). The marked bed bugs included yellow (10), green (6), red (9) (Apple Barrel, Plaid Enterprises, Inc., Norcross, GA, USA) or white (16) (Folk Art, Plaid Enterprises Inc., Norcross, GA, USA) (Fig 1). A single dab of paint was applied to the top of the thorax-abdomen using a fine bristle paint brush. Once the paint was dry, the marked bed bugs were transferred into a round plastic container (4 × 5 cm). The same day both groups (marked and unmarked) were fed defibrinated rabbit blood as described in the previous laboratory bioassay. The next day, the marked and unmarked bed bugs were placed in plastic arena (80 × 75 × 5 cm) lined with brown paper and a layer of fluoropolymer resin applied to inner walls to prevent the bed bugs from escaping. Eight folded paper harborages were placed along the edges in each arena. The arena was kept in a room at 24 ± 1°C, 40% RH, and a 12:12 h (L:D) photoperiod. Mortality was recorded daily over the next 14 d.

### Field Experiments

#### 1. Selection of study apartments

M-r-r experiments were conducted in bed bug infested apartments located in affordable housing communities in three cities in New Jersey: Irvington, Hackensack, and Newark. In each of the three communities, apartments with large bed bug populations were identified by the housing staff and then visually inspected by three Rutgers researchers to assess the extent of the infestation. A total of six apartments (2 vacant, 4 occupied) were selected for m-r-r experiments. All of the apartments were concrete pre-caste construction with the exception of one apartment (referred to later as apt. #5) which was wood frame construction. The criteria for inclusion of occupied units in the study were: 1) residents were not suffering bite symptoms or being negatively affected by the presence of bed bugs in their apartments, and 2) residents agreed with the study procedures. Neighboring apartments above, below, to both sides, and across the hallway from m-r-r apartments were also inspected and monitored for bed bug activity throughout the study. A general description of the six apartments is provided in Table 1. Five of the six apartments were observed for one month between the months of December and February, while one apartment (apt. #6) was observed for five months from December through April.

**Table 1:** Overview of the mark-release-recapture apartments.

**Site**	**Apt. #**	**Apt. size**	Occupancy status	Mean (min, max) temperature inside apt. (°C)^a^	# of interceptors	Pre-count^b^	Number of bed bugs released by area				
							Bedroom^c^		Living room	Bathroom	Total
I	1	Studio (27 m^2^)	Occupied	30 (15–33)	24	305	170 (100:70)^d^	170 (100:70)	170 (100:70)	170 (100:70)	680 (400:280)
I	2	1 BR (45 m^2^)	Occupied	25 (19–29)	27	55	92 (52:40)	92 (52:40)	46 (26:20)	46 (26:20)	276 (156:120)
I	3	1 BR (45 m^2^)	Occupied	23 (13–27)	29	37	40 (20:20)	0	20 (10:10)	20 (10:10)	80 (40:40)
II	4	1 BR (45 m^2^)	Occupied	24 (17–29)	36	105	150 (75:75)	150 (75:75)	150 (75:75)	150 (75:75)	600 (300:300)
II	5	Studio (27 m^2^)	Vacant	_^e^	26	191	180^f^ (90:90)		180 (90:90)	0^g^	360 (180:180)
III	6	1 BR (47 m^2^)	Vacant	23 (18–41)	28	575	159 (90:32:37)^h^		159 (90:32:37)	159 (90:32:37)	477 (270:96:111)

^a^ Temperatures were recorded every hour during the study period using HOBO data loggers (Pendant temp/light, Onset Computer Corp., Bourne, MA.).

^b^ Pre-count for apartments #1, 2, 3, and 6 are based on a 1 d trapping period, apartments #4 and 5 are based on the daily average of a 2 and 4 d trapping period, respectively.

^c^ Numbers in first and second columns refer to bed bugs released at the head and foot of the bed respectively.

^d^ Numbers in parenthesis refer to adult males:adult females.

^e^ Data not available.

^f^ In apartments #5 and #6, marked bed bugs of one color were released along base of wall in the bedroom.

^g^ No bed bugs were released in the bathroom because it was located less than 2 meters from the apartment entry door.

^h^ Numbers in parenthesis refer to large nymphs:adult males:adult females.

1.1 Mark-release-recapture procedures at site I. Apartment #1 was located on the 4^th^ floor of an 11-story high-rise, while apartments #2 and #3 were located in a second 11-story high-rise on the same property. Apartments #2 and #3 were located in opposite wings of the building, on the 8^th^ and 9^th^ floors, respectively. All three apartments had a limited amount of furniture and were not cluttered. In apartment #1, the mattress and box spring were still wrapped in the original plastic from the time of purchase. None of the apartments had been treated for bed bugs prior to the study.

Bed bug adults were collected from each apartment over a two day period. Nymphs were not used for marking because they will molt and lose paint marks. Collection methods included: 1) hand removing using featherweight soft forceps (BioQuip Products, Rancho Dominguez, CA, USA), and 2) placement of two un-baited dog bowl traps [24] beneath the bed overnight. The collected bed bugs were placed in plastic containers (4.7 cm height and 5 cm diameter) with paper harborages and held in the laboratory at 22–25°C and natural light conditions. The bed bugs from each of the apartments were marked 24–48 h after collection. Marked bed bugs were held for a total of two to three days without feeding before being released back into the apartment they were collected from. Any marked bed bugs that were unable to crawl when gently prodded were considered dead or moribund. These bugs were removed and replaced with marked bed bugs of the same color. Total mortality and morbidity of marked bed bugs prior to release was ≤ 1.5% for all three apartments.

**Fig 2:**
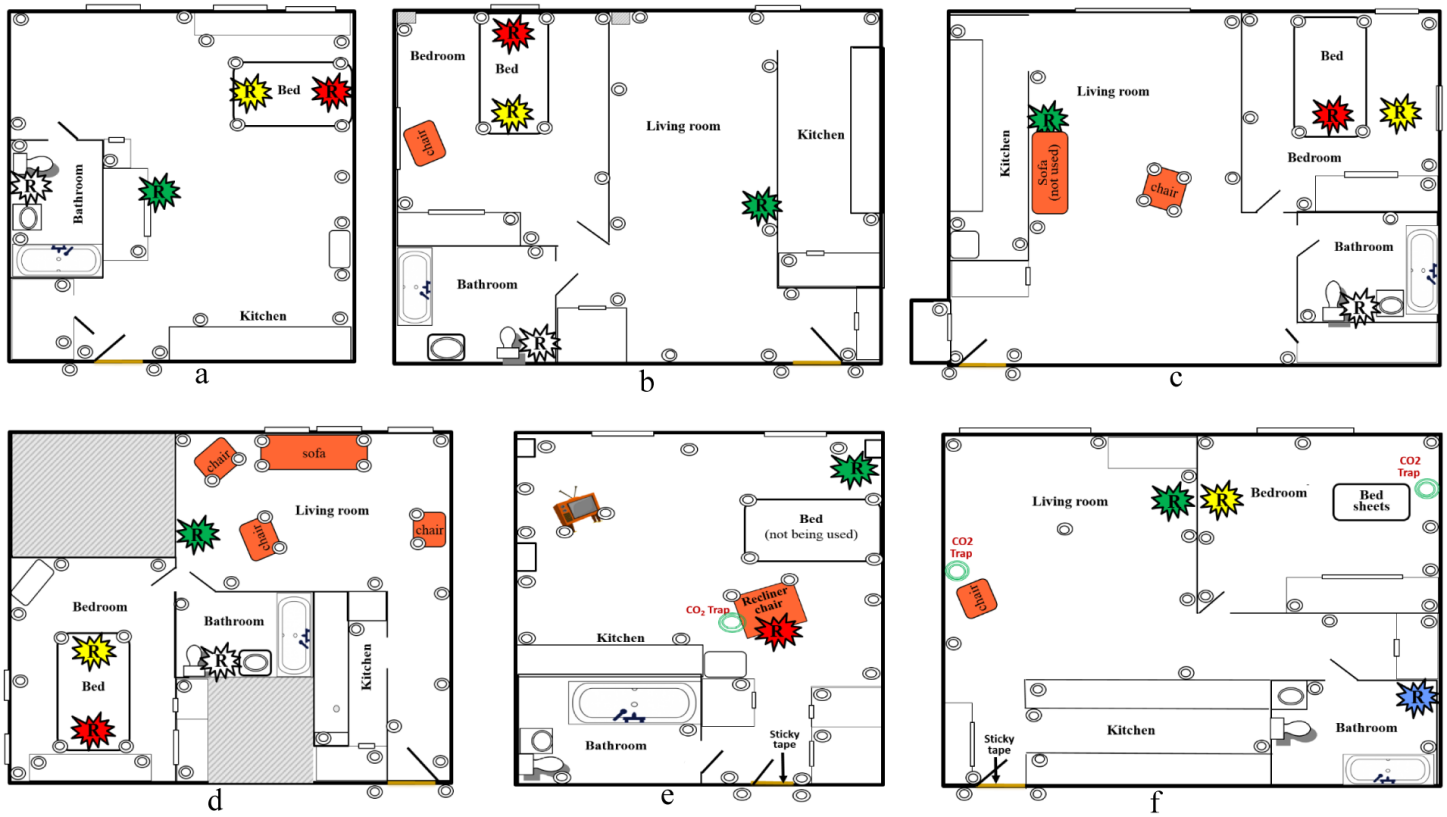
Apartment diagrams and interceptor locations. Letters a to f refer to apartments #1 to 6, respectively. Circles indicate interceptor trap locations. Colored symbols with an “R” inside, indicate where marked bed bugs of a particular color were released.

At the time of the initial collection of bed bugs from the m-r-r apartment, interceptors were installed throughout each of the m-r-r apartments to monitor the bed bug activity. Interceptors were placed next to the legs of the bed frame, rather than beneath the legs. This was done to permit movement of marked and unmarked bed bugs to and from the beds. Interceptors were also placed throughout the apartment in the bedroom, living room, closets, bathroom, and kitchen (Table 1 and Fig 2a–2c). The mean (min, max) distance between the interceptors in the neighboring apartments was 1.28 (0.30, 2.90) m. Two interceptors were also placed in the hallway outside the apartment on either side of the entry door and were secured to the floor with tape to prevent them from being accidentally moved. Interceptors were installed throughout each of neighboring apartments above, below, to both sides, and across the hallway from each of the m-r-r apartments in a similar manner as the m-r-r apartments. The mean (min, max) number of interceptors in the neighboring apartments was 20 (13, 27).

Marked bed bugs were released into the same apartment from which they were collected, and released by removing harborages from a container with bed bugs of the same color and placing them as a group in one location in the apartment. Any marked bed bugs not resting on a harborage, were gently removed from the container with soft forceps and placed on a paper harborage with the other marked bed bugs of the same color. In all three apartments bed bugs were released at the following locations: 1) on the bed beneath the fitted sheet; 2) in the living room on the floor along the wall; and 3) in the bathroom behind the base of the toilet (to reduce visibility). The number of marked bed bugs released in each apartment as well as the release locations are summarized in Table 1 and Fig 2a–2c.

The m-r-r apartments were visited on 1, 3, 6, 10, 15, and 29 d after release. During each of these visits interceptors were inspected. Trapped bed bugs were categorized as either small (1^st^– 2^nd^ instar) or large (3^rd–^ 5^th^ instar) nymphs, and adults were sexed. Captured bed bugs were removed from the interceptors and either destroyed or returned to the laboratory. During each visit, interceptor traps were cleaned and lubricated with talc or replaced with new traps depending upon their conditions. In accordance with the approved IRB protocol bed bug infestations in the three occupied m-r-r apartments were treated at two weeks after the trap catch was recorded.

The neighboring apartments were visited on 3, 6, 10, 15, and 29 d during which time interceptors were inspected and trap catch recorded as described above for the m-r-r apartments. A visual inspection of beds and upholstered furniture was conducted during the final visit.

1.2 Mark-release-recapture procedures at site II. Apartment #4 was located on the 3^rd^ floor of a 7-story building and apartment #5 was located on the 4^th^ floor of a 10-story building. The bedroom of apartment #4 was cluttered. The mattress, box spring, bed frame, and built-in headboard were heavily infested. The living room was furnished with a sofa and two upholstered chairs, all with signs of bed bug infestation, but no live bed bugs were observed during visual inspection. The apartment was treated with a liquid residual pyrethroid, Suspend SC (0.03% deltamethrin, Bayer Environmental Science, Montvale, NJ, USA) by the existing pest control contractor less than one hour prior to our initial inspection, however they did not remove the mattress and box spring during their treatment. Adult bed bugs were collected from the mattress and box spring and returned to the laboratory in the same manner as described for apartments at site I. The bed bugs were held for three days prior to marking. Their mortality was 1.1%, suggesting that the bed bugs were either not contacted by the insecticide or were resistant to the pyrethroid insecticide applied. After being marked, they were held in the laboratory for two more days and dead or moribund bed bugs were replaced with freshly marked bed bugs immediately prior to release. The mortality among marked bed bugs was < 1.0% prior to release.

Prior to releasing the marked bed bugs, interceptors were installed in the same manner as described for site I (Table 1 and Fig 2d). The mean (min, max) distance between the interceptors in the neighboring apartments was 1.28 (0.30, 2.90) m. The neighboring apartments of # 4 were also monitored prior to the release of marked bed bugs, in the same manner described for site I. The mean (min, max) number of interceptors per neighboring apartment was 29 (25, 31).

Marked bed bugs were released in the same manner as described for site I (Table 1 and Fig 2d). The apartment was visited on 1, 3, 7, 14, 21, and 28 d and inspected as described for site I. At 14 d after the trap catch was recorded the apartment was treated in accordance with the approved IRB protocol for occupied units. The neighboring apartments were visited following the same schedule as the m-r-r apartment.

The resident in apartment #5 was evicted the day the m-r-r study began. The apartment was severely cluttered with piles of trash, papers and clothing. Furniture in the apartment was limited to a bed, TV stand, dresser, and recliner. The resident had slept in the recliner for several months prior to the start of the study. Bed bugs were hand collected the same day the resident was evicted. In addition, a CO_2_ trap [24] was placed overnight at the recliner to collect more bed bugs. CO_2_ was released from the trap at a rate of 200 ml/min between the hours of 10 pm and 8 am. Bed bugs were collected from the CO_2_ trap after one day and were held in the laboratory for 24–48 h before being marked. Dead bed bugs were replaced with freshly marked bed bugs immediately prior to release. Mortality of the marked bed bugs prior to release was < 1.0%. Prior to releasing the marked bed bugs, interceptors were installed (Table 1 and Fig 2e). The mean (min, max) distance between the interceptors in the neighboring apartments was 1.16 (0.30, 2.90) m. In addition to the interceptors, a 7.6 cm wide sticky tape barrier was installed across the inside threshold of the entry door to intercept bed bugs traveling at the base of the door. Interceptors were installed in the neighboring apartments six days prior to the release of marked bed bugs, in the same manner described for site I. The mean (min, max) number of interceptors per neighboring apartment was 27 (20, 31).

The CO_2_ trap used for the collection of bed bugs remained in place to stimulate bed bug activity in the absence of a host during the monitoring period. The trap was set to release CO_2_ at 200–400 ml/min between the hours of 10 pm and 8 am and was turned off at 1, 8–9, 22–23, and 26–28 d to investigate the influence of CO_2_ on bed bug movement. The same day the monitors were installed, property management bagged and removed all clutter from the apartment leaving only the bed, reclining chair, wooden dresser, and TV stand.

Marked bed bugs were released in two locations: 1) on the recliner 0.5 m from a CO_2_ trap, and 2) along the base of the wall next to the bed (Table 1 and Fig 2e). No marked bed bugs were released in the bathroom because it was located immediately adjacent to the entry door and we did not want to promote dispersal so close to the apartment entry. The apartment and its neighbors were visited on 1, 3, 7, 14, 21, 28, and 35 d. The sticky barrier was replaced at least once per week and CO_2_ cylinders were replaced as needed to prevent running out before the next visit. At 35 d the apartment was treated for the first time, it had not previously been treated prior to the start of the study.

1.3 Mark-release-recapture procedures at site III. Apartment #6 was located on the 5^th^ floor of a 5-story apartment building. The apartment had become vacant 17 d prior to the m-r-r study. Furniture in the apartment was limited to an upholstered chair in the living room along with a small coffee table and a TV. Clothing, boxes, and piles of papers were strewn about the living room. The bedroom had a wooden folding chair and blankets on the floor that the resident slept on. The blankets were heavily infested. Bed bugs were present along the base of the wall less than one meter from where the resident slept. The apartment had not been treated for bed bugs prior to the m-r-r study.

Bed bug adults and large nymphs were hand collected using soft forceps and two CO_2_ traps over a two day period. We collected large nymphs at this site because the apartment had been vacant for 17 d prior to collection, thus no more nymphs would molt. They were marked 24–48 h after collection and held for another two days before being released. Dead bed bugs were replaced with freshly marked bed bugs immediately prior to release. Mortality of the marked bed bugs prior to release was <1.5%. One day prior to release, interceptors were installed in the m-r-r apartment and a sticky tape barrier was installed at the entry door as described for apartment #5 (Table 1 and Fig 2f). Marked bed bugs were released in the same manner as for site I. Interceptors were also installed in its four neighboring apartments in a similar manner as previously described for site I. The mean (min, max) number of interceptors per neighboring apartment was 30 (27, 33).

The two CO_2_ traps, used for the collection of bed bugs were left in place during the post-release monitoring period and maintained in a similar manner as apartment #5. The CO_2_ was turned off at 2–4, 6–9, and 18–21 d to investigate the influence of CO_2_ on bed bug movement. The apartment was visited daily during the first 12 d, every 3 d for the next 18 d, and then every 5–7 d through 116 d. Interceptors were inspected and maintained as described for site I. On the 24^th^ d the contents of the apartment (furniture, clothing, and debris) were bagged and discarded leaving the apartment empty aside from the monitoring devices. Alpine dust (0.25% dinotefuran, 95% diatomaceous earth dust, Whitmire Micro-Gen Research Laboratories, St. Louis, MO, USA) was applied to the baseboards throughout the apartment. At 71 d, the CO_2_ traps were removed from the apartment for the remainder of the study. The interceptors in the neighboring apartments were inspected at 3, 5, 12, 19, and 24 d post-release.

### Estimating Population Size

The following conditions are required for estimating populations using m-r-r method [27]: 1) the marked bed bugs retain their marks; 2) the marked bed bugs mix thoroughly with the rest of the population; 3) a sample representative of the whole population is taken for marking and for estimation; 4) the population is closed, or rates of immigration and emigration are known; and 5) there are no births or deaths during the period of mixing. Our study did not meet the last two requirements. However, because we were only sampling for a short period of time, we could assume the population size in each apartment was relatively stable.

The bed bug population size in each apartment was estimated using Peterson-Lincoln index [27]: N = M(C+1)/(R+1), where N = total population, M = number of marked bed bugs released, and C = total number in the re-capture sample (marked + unmarked), R = the number of marked individuals in the recapture sample. The variance is calculated by the formula: V = M^2^(C-R)/((C+1)(R+1)). Adult stage was used in the population estimates because nymphs were only marked for one of the six m-r-r apartments. The estimated bed bug population is calculated as the estimated number of adults divided by the proportion of adults among all trapped bed bugs.

### Statistical Analyses

The effect of marking procedure on bed bug survival was analyzed using a chi-square test. Percentage of bed bugs of various stages or sex trapped in interceptors, recapture rate of marked bed bugs, the small:large nymph ratio at the bed and apartment entry, and the male:female ratio of released and recaptured marked bed bugs were subjected to Student’s *t* test. The effect of CO_2_ presence on trap catch in a vacant unit was examined using analysis of variance. Correlation analysis was conducted between the number of dispersed and recaptured bed bugs and the number of released bed bugs. All field data analyses were based on interceptor trap catch over 14–15 d prior to treatment with pesticides. All analyses were performed using SAS software version 9.3 (SAS Institute, Cary, NC, USA).

## Results

### Laboratory bioassays

#### 1. Reliability of interceptors for estimating bed bug population structure

Among the trapped bed bugs in interceptors, the percentage (mean ± SEM) of 1^st^ instar, 3^rd^-5^th^ instar, adult males, and adult females was 21.8 ± 2, 22.7 ± 2, 25.7 ± 1, and 29.8 ± 1%, respectively (Table 2). There was a significant bias for trapping adult females (t = 2.6; df = 5; *P* = 0.046). The mean percentage of females in the trapped population was 28% more than the percentage of females in the population initially released in the arena, indicating females moved more than adult males and nymphs. The mean percentage of all other stages trapped was similar to the percentages released (1^st–^ 2^nd^ instar: t = -0.7, df = 5, *P* = 0.50; 3^rd^– 5^th^ instar: t = -0.3, df = 5, *P* = 0.81; males: t = -0.9, df = 5, *P* = 0.40), indicating that the interceptor trap catch is not biased for nymphs and adult males.

**Table 2:** Trap and escape of bed bugs from interceptors.

Rep.	Number of bed bugs trapped in interceptors in 4 days					Number of bed bugs escaped from interceptors within 10 days				
	Adult males	Adult females	1^st^ instar nymphs	3^rd^-5^th^ instar nymphs	Total	Adult males	Adult females	1^st^ instar nymphs	3^rd^-5^th^ instar nymphs	Total
1	14	22	15	13	**64**	0	0	1	0	**1**
2	22	22	18	14	**76**	0	0	3	0	**3**
3	17	20	9	16	**62**	0	0	3	0	**3**
4	19	20	10	20	**69**	0	0	4	0	**4**
5	18	20	20	16	**74**	0	0	4	0	**4**
6	23	26	26	20	**95**	0	0	1	0	**1**
Sum	113	130	98	99	**440**	0	0	16	0	**16**
Mean	19	22	16	17	**73**	0	0	2.7	0	**2.7**

Only 1^st^ instar bed bug nymphs escaped from interceptors (Table 2). The mean ± SEM percentage of escape in 10 d was 20 ± 6%. Overall, among all stages of bed bugs trapped, the mean ± SEM escape rate in 10 d was 4 ± 1%. The mean ± SEM mortality among bed bugs still trapped in interceptors at 10 d was 99 ± 1%.

#### 2. Effect of marking procedure on bed bug survival

**Fig 3:**
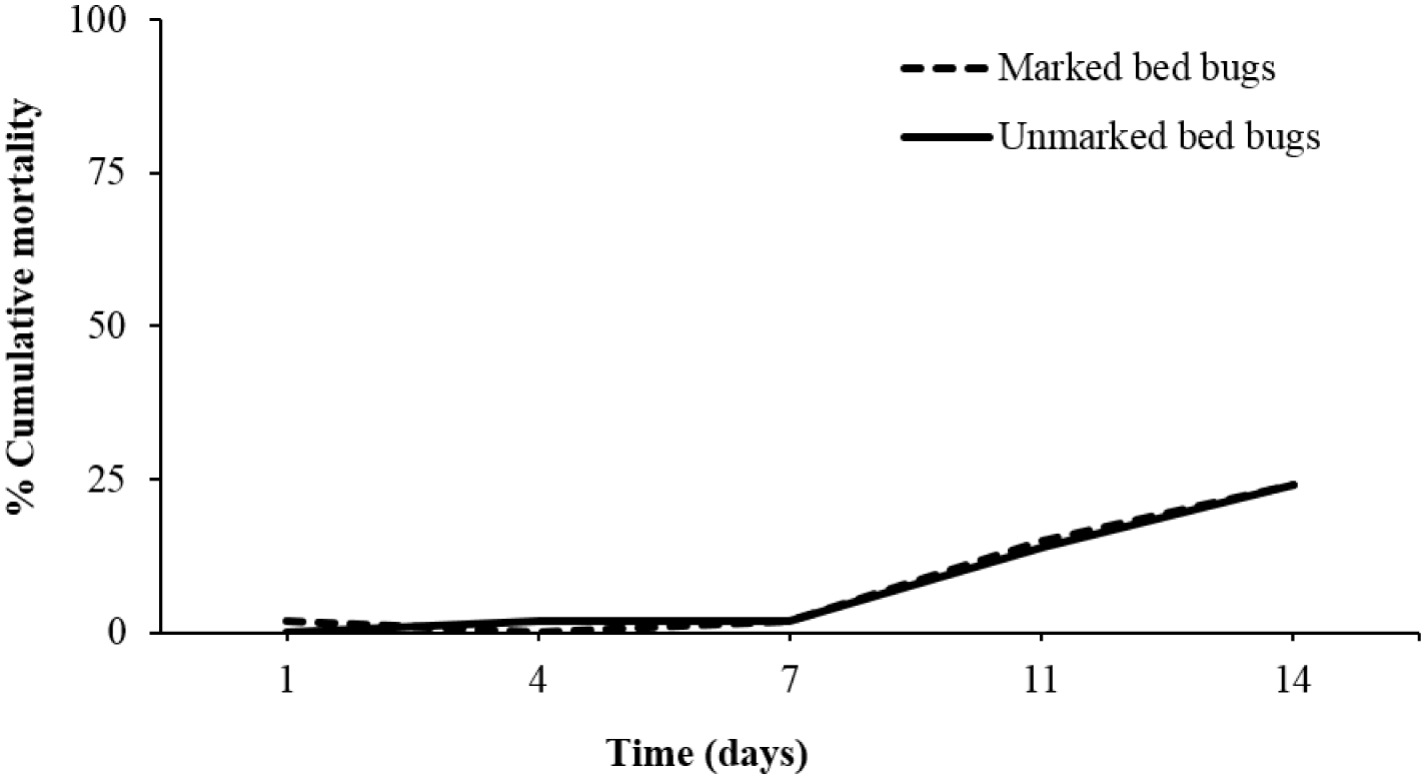
Cumulative mortality of marked and unmarked bed bugs under laboratory conditions.

Cumulative mortality over 14 d was 24% for both marked and unmarked bed bugs (Fig 3). There was no significant difference in the mortality of marked (14%) and unmarked (13%) males (χ^2^ = 0.013; df = 1; *P* = 0.91) or marked (37%) and unmarked (36%) females (χ^2^ = 0.003; df = 1; *P* = 0.95). The results indicate that the marking procedure did not have an effect on the survival of the bed bugs. However, female mortality (marked and unmarked) was higher than male mortality (marked and unmarked) (χ^2^ = 5.13; df = 1; *P* = 0.02).

### Field Experiments

#### 1. Movement within apartments

Marked bed bugs were captured in interceptors located at and away from release locations at 24 h post-release in all six m-r-r apartments (S1 Fig). Recapture rates for the marked bed bugs released in the six m-r-r apartments ranged from 6–72% by 14–15 d (Table 3). The two vacant apartments had the highest recapture rates (Table 3). Females represented 48% ± 2% of the marked adult bed bugs released and 54% ± 1% of the marked adults recaptured. The percentage of recaptured females was significantly higher than the expected percentage (t = 4.1; df = 5; *P* = 0.01). Therefore, the counts based upon interceptors were biased towards females, suggesting females were more active than males. Similarly, there were significantly more females than males among the 1,810 unmarked adults captured in the m-r-r apartments (t = 3.56; df = 5; *P* = 0.02). The mean (min, max) number of marked and unmarked bed bugs captured in interceptors at the entry door over 14–15 d from all six m-r-r apartments was 3.5 (1, 11) and 37.2 (1, 74), respectively. The sticky tape barrier captured 9 marked bed bugs and 248 unmarked bed bugs of all stages in apartment #5, and 2 marked bed bugs and 21 unmarked bed bugs of all stages in apartment #6. These results indicate that bed bug activity is prevalent at apartment entries.

**Table 3:** Cumulative recapture rate of marked and released bed bugs after 14 days.

Apt.#	Total recapture rate	Recapture rate by stage and adult sex			Recapture rate by release site^a^		
		Large nymphs	Male	Female	Bedroom	Living room	Bathroom
1	40%	‒^b^	31%	54%	37%^c^		52%
2	31%	‒	25%	38%	18%	76%	37%
3	28%	‒	25%	30%	5.0%	60%	40%
4^d^	6%	‒	6%	7%	7%	5%	‒
5	72%	‒	64%	79%	72%		‒
6	44%	50%	31%	38%	48%	45%	39%

^a^ This rate is the number of bed bugs released in a given room that were recaptured throughout the apartment divided by the number released in that room.

^b^ No marked bed bugs were released or the resident discarded harborages with marked bed bugs on the day of release (apt. #4).

^c^ Studio units (#1 and 5) had no distinction between the bedroom and living room.

^d^ Resident interfered with study by moving and emptying interceptor traps.

Based on four apartments (#1, 2, 3, and 5) where bed bugs were released directly on the furniture used by the resident to sleep, between 38–67% of the recaptured marked bed bugs were in interceptors located at least 2.5 m from the host sleeping area. Among the three one bedroom apartments (#2, 3, and 6), recapture rates were significantly greater in the room of release than the non-release rooms, when marked bed bugs were released in either the bedroom (F = 9.0; df = 3, 8; *P* = 0.01) or living room (F = 7.9; df = 3, 8; *P* = 0.01), but not the bathroom (F = 2.8; df = 3, 8; *P* = 0.11) (Table 4). Of those released in the bedroom of these three apartments, a mean of 42 ± 7.5% of the marked bed bugs were recaptured outside of the bedroom. The mean ± SEM percentage of unmarked bed bugs captured at the bed, in the bedroom away from the bed, in the living room, in the bathroom, and in other areas (i.e. hallway, kitchen, entry door) was 15 ± 5, 41 ± 10, 30 ± 9, 3 ± 1, and 11 ± 6%, respectively. These results demonstrate the extensive movement of bed bugs within apartments.

**Table 4:** Movement of marked bed bugs within apartments based on 14 day cumulative trap catch.

Apt.#	Total number of recaptured marked bed bugs	Percentage of marked bed bugs recaptured by location based upon point of release											
		Released in bedroom				Released in living room				Released in bathroom			
		Bed- room	Living room	Bath- room	Other^a^	Bed- room	Living room	Bath- room	Other	Bed- room	Living room	Bath- room	Other
2	85	73	18	6	3	23	71	3	3	24	64	6	6
3	22	50	0	0	50	0	75	0	25	38	25	12	25
6	208	51	21	12	16	24	51	11	14	27	33	22	18
**Mean**	**105**	**58.0**	**13.0**	**6.0**	**23**	**15.7**	**65.7**	**4.7**	**14.0**	**29.7**	**40.7**	**13.3**	**16.3**

^a^ Other areas include the apartment entry door, kitchen, hallway, and closets.

A total of 4,076 (4,062 unmarked and 14 marked) bed bugs were captured in 20 of the 24 neighboring apartments over 14–15 d. Fifteen of the 20 apartments were one bedroom units and the rest were studio units. The median (min, max) bed bug count in the 20 infested apartments was 6.5 (1–3,162) bed bugs over 14–15 d. Bed bugs were captured outside of the bedroom and living room in 18 of 20 apartments. In the three one bedroom apartments with sufficient adult bed bug counts (> 10) for analysis, the mean percentage of females captured in the bedroom and living room compared to areas outside the bedroom and living room was 0.71 ± 0.08 and 0.55 ± 0.12, respectively. They were not significantly different (t = 0.85; df = 2; *P* = 0.48).

#### 2. Movement between apartments

A total of 14 marked bed bugs (7 nymphs, 6 females, and 1 male) were recaptured in neighboring apartments of four m-r-r apartments over 14–15 d (Table 5). Among these four m-r-r apartments, at least one of the dispersing bed bugs in each apartment was released at the host sleeping area. Marked bed bugs of three different colors dispersed from apartment #6. These results indicate that bed bugs from any room within an apartment, even those located at host sleeping sites, have the potential to disperse to neighboring apartments. The highest dispersal rate (the percentage of marked and recaptured bed bugs in neighboring apartments divided by total recaptured marked bed bugs) at 14–15 d was 5.0%. The number of recaptured bed bugs in neighboring units was not correlated with the number of released bed bugs (F = 0.96; df = 1, 4; *P* = 0.38). The fact that one vacant apartment (#6) had the highest active dispersal rate and the other vacant apartment (#5) had no active dispersal indicates that vacancy is not necessarily correlated to bed bug dispersal.

**Table 5:** Active dispersal of bed bugs revealed from m-r-r technique over 14–15 days.

Apt. #	# of unmarked/ marked bed bugs trapped in mark-release apt.	Number of unmarked/marked bed bugs captured in apts. surrounding the m-r-r apts.					% dispersal rate^a^	Areas where marked bed bugs dispersed from	Areas where marked bed bugs were recaptured in neighboring apts.
		Adjacent to the right	Adjacent to the left	Across hall	Above	Below			
1	3,090/280	8/1	3/0	6/0	120/0	37/0	0.4	Bed	Bedroom
2	220/85	3162/1	na	Na	1/0	1/0	1.2	Bed	Kitchen
3	288/22	0/0	0/0	Na	0/0	7/0	0	None	None
4	1,020/30	575/1	87/0	na.	1/0	0/0	3.2	Bed	Kitchen
5	11,315/258	2/0	na	2/0	5/0	1/0	0	None	None
6	1,924/208	26/2	27/4	7/4	na	3/1	5.0	Bedroom, living room, bathroom	Kitchen, hall, living room

^a^ Dispersal rate is calculated as the total number of marked bed bugs recaptured in neighboring apartments divided by the total number of marked bed bugs recaptured.

Between 16–28 d, marked bed bugs dispersed from two m-r-r apartments (#4 and 5), one of which (apt. #5) did not show active dispersal during the first 15 d. Each of these two apartments had two marked adult females captured in their neighboring apartments. It should be noted that apartment #4 was treated at 15 d. Additionally, a marked adult female (green) was captured in an interceptor in the hallway outside of apartment #2. Although active dispersal was not recorded for apartment #3, two marked adult females, one released at the bed (yellow) and one released in the living room (green), were captured in interceptors at the entry door of this apartment. Over 28–32 d a total of 12 marked bed bugs (11 females, 1 male) were recaptured in ten neighboring apartments. All of these neighboring apartments had bed bug activity. Horizontal dispersal of marked bed bugs to a neighboring apartment on the same floor occurred among all four m-r-r apartments where dispersal was recorded within 28–32 d. Marked bed bugs were found in two of the three apartments that were across the hallway from a m-r-r unit. Vertical dispersal, on the other hand, was observed from two out of the five m-r-r apartments.

#### 3. Longevity in the absence of a host

In apartment #6, the mean (min, max) daily temperature inside the apartment during the study was 23 (18, 41)°C. After release, marked large nymphs, adult females, and adult males continued to be captured up to 57, 113, and 134 d after vacancy, respectively.

**Fig 4:**
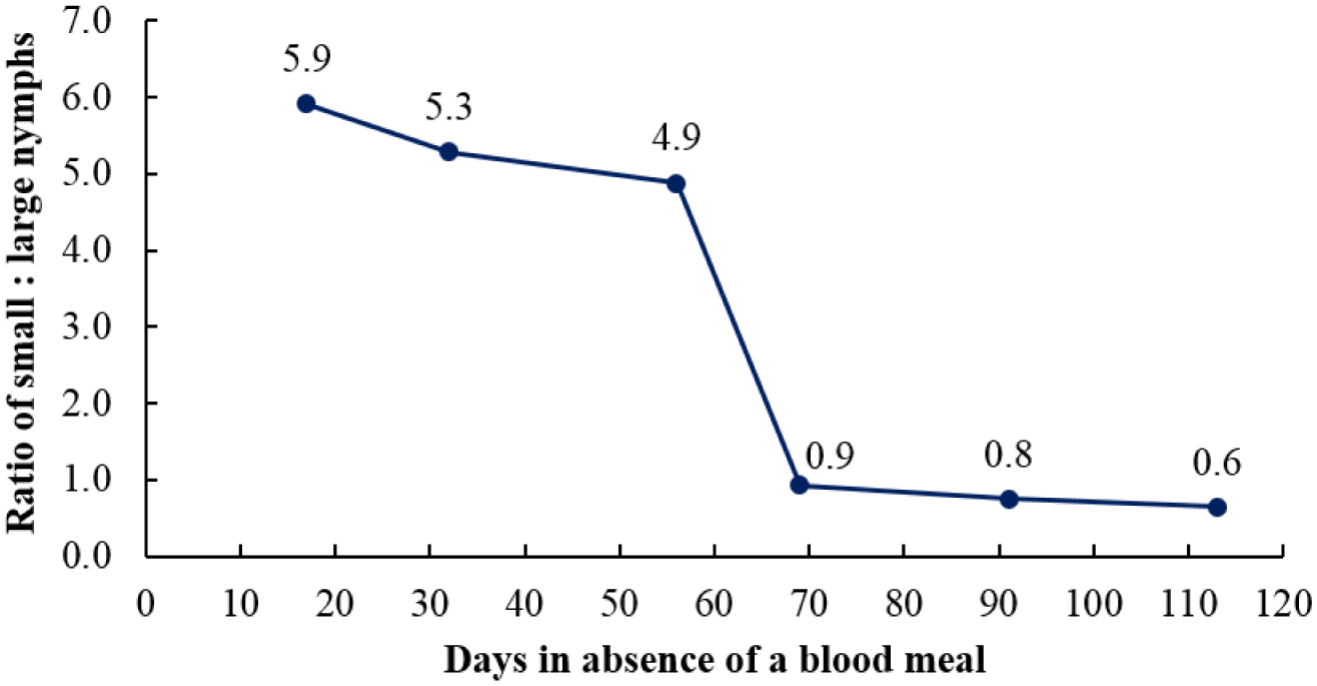
Ratio of unmarked small:large nymphs over time in the absence of a host in a vacant apartment (apt. #6).

Unfed, unmarked small nymphs were no longer captured in interceptors after 134 d. Other stages of unfed bed bugs continued to be found in interceptors after 134 d. When the experiment was terminated at 155 d, 16 unmarked bed bugs were captured in interceptors over a 7 d trapping period. Of these, seven were large nymphs and nine were adults (8 female, 1 male). These results demonstrate that young nymphs were able to survive at least 4.5 mo and all other stages over 5 mo in the absence of a host. The small:large nymph ratio declined sharply after 56 d (Fig 4). The sudden decline suggests that most small nymphs survived starvation for 52–69 d and large nymphs are more tolerant to starvation than small nymphs.

#### 4. Estimating population size

Four apartments (#1, 2, 5, and 6) were selected to estimate population size. The other two apartments (#3 and 4) had too few recaptured marked bed bugs (4 and 7 bugs) during 4–7 d and therefore were excluded from population estimation analysis.

**Fig 5:**
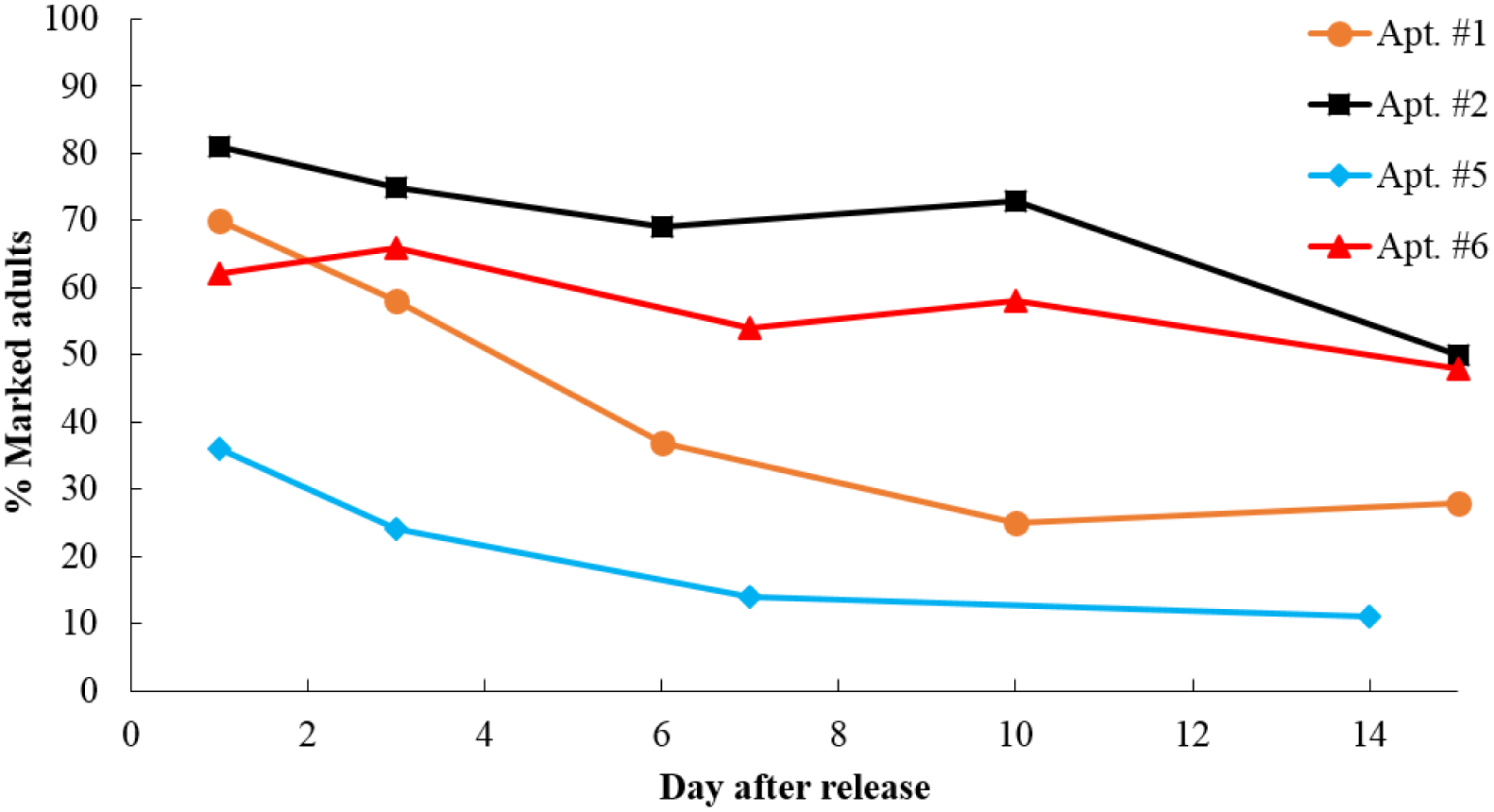
Dynamics of the ratio of marked adult bed bugs over all adult bed bugs captured from interceptors.

We used data where the percentage of marked adults became stable (4–7 d) to estimate bed bug populations. The percentage of marked bed bugs in the interceptors was high during the first 3 d after release and became relatively stable until 10 d (Fig 5). The initial high percentage of marked adult bed bugs was expected, as the released bed bugs might need a few days to acclimate. The M in the population estimate formula (N = M(C+1)/(R+1) was estimated as the number of bed bugs initially released minus the recaptured marked bed bugs during the first three days because these bed bugs were no longer present. The population estimates for each of the four apartments are summarized in Table 6.

**Table 6:** Bed bug population estimation based on m-r-r technique.

Apt. #	Trapping period	Total # of marked adult bed bugs existed at 4 d^1^	Proportion of marked adult bed bugs^2^	Estimated total adults	Proportion of adults in unmarked bed bugs	Estimated total population	Standard deviation
1	4 to 7 d	505	0.3778	1,337	0.09354	14,291	1,578
2	4 to 6 d	226	0.7059	320	0.13158	2,433	381
5	4 to 7 d	177	0.1396	1,268	0.10304	12,305	1,630
6	4 to 7 d	173	0.5476	316	0.02794	11,306	1,586

^1^ This is the number of bed bugs initially released minus the recaptured marked bed bugs during the first three days.

^2^ This is the (R+1)/(C+1) used in the formula for population estimation.

Once the estimated population size is available, it is possible to estimate what percentage of bed bugs from an apartment can be caught in an interceptor during one day. We used the mean daily counts per interceptor during 4–7 d from four interceptors installed in the area used by the host to sleep during the night. We selected interceptors in these locations to demonstrate how effective interceptors are because this is where interceptors are typically placed by users. Daily trap catch data were only available in one of the two vacant apartments (#6) over the 4–7 d period. During this time CO_2_ was released for 1 d and then turned off during the other days. When CO_2_ was released, the estimated percentage of bed bugs trapped per interceptor, per day was 0.55%, and when CO_2_ was absent it was 0.11 ± 0.02%. Therefore, adding a CO_2_ source can greatly increase the trap catch. In the two occupied apartments (#1 and 2), 0.07 and 0.61% bed bugs were trapped per interceptor per day.

The effect of a CO_2_ source on interceptor trap catch was further demonstrated in apartment #6. The daily total catch from 28 interceptors were recorded when CO_2_ was not available during 7–9 d and when CO_2_ was released daily at 200–400 ml/min for 10 h per day during 10–12 d. The mean daily total catch was 29 ± 7 and 111 ± 26, respectively. They were significantly different (F = 9.6; df = 1, 4; *P* = 0.04). The mean trap catch when CO_2_ was released was 3.8 × higher than when no CO_2_ was released.

## Discussion

Placement of pitfall-style interceptor traps throughout apartments in conjunction with a m-r-r method, proved effective for investigating the activity of *C*. *lectularius* under field conditions. Our results demonstrate that bed bugs of all developmental stages travel extensively within and between apartments within a building. The dispersal rate of marked bed bugs to neighboring apartments was not correlated to the number of bed bugs released. Starved bed bugs can survive for more than five months under field conditions. These findings provide new insights into the behavioral ecology of bed bugs within infested apartments.

Wang et al. [9], [14] showed that over 55% of the bed bugs captured in traps placed under furniture legs were traveling to the beds and upholstered furniture rather than originating from the host sleeping areas. Our results confirm that bed bugs travel extensively within infested apartments in areas away from host sleeping and resting sites. Within 24 h of release, marked bed bugs were captured both at and away from host sleeping/resting areas regardless of their release location in all five m-r-r apartments. Over the course of 14–15 d, between 39–67% of the marked bed bugs released on the furniture where the resident slept during the night traveled at least 2.5 m from the host sleeping area before being captured and 42% of the marked bed bugs released in bedrooms were recaptured outside of the bedroom. The movement of marked bed bugs away from host sleeping areas demonstrates the extensive movement of bed bugs within infested apartments.

It is possible that the movement of the marked bed bugs observed in our study included an artifact created by our experimental design, which involved relocating many of the marked bed bugs to areas other than where they were collected. However, the unmarked bed bugs captured in interceptors represents the natural activity of the population in the m-r-r apartments. Among the unmarked bed bugs captured, 14% were found in interceptors located in bathrooms, kitchens, hallway closets, and door entry areas. In a case study of two occupied homes, bed bugs were marked in situ at resting locations [12]. Similar to our results, marked bed bugs moved from host sleeping areas to non-sleeping areas, from non-sleeping areas to different non-sleeping areas, from non-sleeping areas to host sleeping areas, and were recaptured up to nine meters away from their original resting locations. The authors concluded that bed bugs actively move throughout infested dwellings [12].

The active dispersal of bed bugs from an infested apartment to neighboring apartments has long been suspected [14], [16–19], [21–22], but never proven. Using the m-r-r technique, we were able to definitively demonstrate active dispersal of bed bugs between apartments. Active dispersal to one or more neighboring apartments was confirmed among five of the six m-r-r apartments. Active dispersal occurred from both vacant as well as occupied apartments and was not correlated the number of marked bed bugs released. Moreover, the highest, and one of the lowest, active dispersal rates were observed in the two vacant apartments. The wide variability in the degree of active dispersal among the m-r-r apartments indicated that factors other than infestation level and host availability affect the active dispersal rate.

Active dispersal of bed bugs between apartments has been used as an explanation for infestation clusters and the spread of bed bugs in multi-occupancy buildings [14], [18], [28]. For example, Doggett and Russell [20] documented the spread of bed bugs from 1 to 68 living units in a high rise housing facility in just 25 mo. Among the infested units 85% shared a common wall, ceiling, floor, or were across the hallway from another unit with bed bugs [20]. In our study, among the six m-r-r apartments 83% of the 24 neighboring apartments had bed bug activity. A number of our findings suggest that active dispersal may be the primary cause for these infestations: 1) 92% (11 of 12) of the marked adults recovered in neighboring apartments were females; 2) marked bed bugs were found in 50% of the 20 neighboring infested apartments; 3) marked bed bugs dispersed from the m-r-r apartment to apartments in all directions (above, below, adjacent, and across the hallway); and 4) the m-r-r units had higher population levels than the neighboring infested units except for two apartments. Current recommendations for neighboring unit inspections in multi-occupancy dwellings include the units above, below, and adjacent to the known infested unit [29]. However, based upon our findings we recommend expanding the scope of neighboring unit inspections to include units located across the hallway from known infestations.

Recent studies have used aggregation behavior to draw conclusions about dispersal [16], [18–19]. A limitation of this approach is that it doesn’t consider the temporal activity of the bed bugs moving between aggregations and resting sites. In contrast, our approach relies upon intercepting the movement of bed bugs (marked and unmarked) as they travel within the infested dwelling, over time. However, there are also limitations associated with this approach: 1) trapped bed bugs are prevented from reaching their destination and therefore their potential to spread is reduced; 2) because bed bugs were not marked in situ within individual aggregations, we altered the natural distribution and are also likely to have increased their movement.

While our study does not answer the question as to why bed bugs are dispersing, it does provide important insight that can serve as the basis for future study on dispersal mechanisms. For example, it has been suggested that adult females may disperse to avoid the deleterious effects associated with repeated traumatic insemination by adult males [16–17], [30], [31] and in doing so, can expand the population range and create new infestation sites [16], [22]. Pfiester et al. [16] also suggested that following female dispersal, male bed bugs may also begin to disperse in search of females. Laboratory bioassays conducted by Naylor [18] demonstrated that adult male and female bed bugs disperse in equal numbers, a finding which was supported by observations that were made at a single field site. However, we found among marked and unmarked adults, a greater number of females were captured in interceptors, and nine of the ten marked adults that actively dispersed from m-r-r apartments to neighboring apartments were females, demonstrating that females are more active and travel farther than males. Whether or not female dispersal is based upon avoidance of males is unclear.

Another commonly held belief is that bed bugs disperse as infestation levels increase [12], [18], [28]. Potter et al. [12] observed dispersal of marked bed bugs away from their original resting sites, but pointed out that the field sites had well established infestations and that the degree of movement might differ with smaller populations. Naylor [18] conducted extensive laboratory experiments on bed bug dispersal and observed bed bug infestations in four buildings concluding that bed bug aggregations were rarely located more than 2.3 m away from where the host slept, particularly in populations of fewer than 100 bed bugs, and that population density within harborages seems to be the main driving force for dispersal. In our study there was no relationship between the active dispersal rate of marked bed bugs to neighboring apartments and the number of marked bed bugs released in the m-r-r apartments. These results suggest that infestation level alone is not responsible for dispersal. Factors such as the amount of clutter and resident behavior may also have affected the dispersal. However, our sample size was not large enough to analyze these relationships.

One possible explanation for the extensive movement of bed bugs away from host feeding sites could be that the bed bugs have become lost and are unable to locate the host at which time they begin to randomly forage in search of a host. Bed bugs orient towards host cues (primarily CO_2_ and heat) over a relatively short distance up to 2 m [32–34] in order to obtain a blood meal. This could explain why Naylor [18] rarely found aggregations located more than 2.3 m away from where the host slept unless infestation levels were severe. It is possible that bed bugs located outside this range have difficulty locating the host. In bed bugs, a negative correlation exists between starvation duration and aggregation behavior [8], [16], [35] and hungry bed bugs have been reported to travel up to 20 m to find a host [7], [36]. Movement of insects away from their harborage sites can increase with hunger, and this may also promote formation of new harborage sites [37–38]. Kemper [36] suggested that for bed bugs, random appetitive searching is important and found that individuals unable to locate a blood meal will continue to forage all night. Reis and Miller [39] also demonstrated continued and prolonged foraging among bed bugs that could not access a blood meal in a laboratory bioassay. Potter et al. [12] recovered marked bed bugs in interceptor traps up to 9 m from where they had been resting 7 d earlier. In our study it was impossible to determine the exact distance that marked bed bugs traveled, however large nymphs and adult females traveled at least 12 m, based upon the shortest path from the point of release to point of capture in apartments across the hallway. Thus it is possible that bed bugs captured away from host sleeping areas in our study represent bed bugs that were unable to locate the host and continued to forage in a random manner in search of a food source, as suggested by Kemper [36]. We did not specifically record the feeding status of the bed bugs captured in interceptors, however based upon our observations, little to no blood remained in the digestive tract of most bed bugs captured in interceptors and capture of engorged individuals was rare.

For an ectoparasite such as the bed bug, that is capable of surviving long periods (several months or more) without feeding, having a portion of the population “searching” for food could serve as an important mode of dispersal. This might also explain how bed bugs locate different host sleeping and resting sites within a home (i.e. multiple bedrooms, living room, family room, finished basements). More broadly dispersed populations within a single dwelling would also be harder to eliminate compared to populations that are limited to a single bed or bedroom, and in multi-occupancy dwellings would promote spread to other living units.

Previous studies examined bed bug starvation tolerance at various temperatures [1], [4], [6], [40]. Early studies by Kemper [1] and Johnson [4] reported first instars surviving 84 and 210–213 d in the absence of a blood meal at 22 and 7°C, respectively. At 13°C a single starved adult female survived 562–572 d [1]. More recently, under laboratory conditions of 26.1–26.5°C, the maximum survival times for starved first instar nymphs, adult males, and adult females were 38, 106, and 99 d, respectively [40]. We continued to capture starved 1^st^ or 2^nd^ instar nymphs in interceptors up to 134 d and all other stages and adults of both sexes were still being captured at 155 d, when the study was terminated. The mean temperature over the 155 d was 23°C, however the temperature fluctuated widely between 18–41°C throughout the study, climbing during the day due to solar radiation from a southerly exposure to the sun through windows that had no curtains. Under a more stable temperature range as is expected in infested dwellings, survival times may be even longer than what we observed. The large population size in the apartment could also explain the long survival times observed. The greater the initial population size, the higher the number of bed bugs that are likely to survive for longer periods of starvation. Additionally, the pesticide resistance profile for the bed bugs in our study is unknown, which could either increase or decrease the survival times observed.

Our laboratory assays show that interceptors can be used to estimate bed bug population structure with slight bias toward females. Only first instar nymphs were able to escape following capture in interceptors. The high mortality rates among trapped bed bugs is similar to what is seen under field conditions (personal observation) and may be the result of desiccation of the exposed bed bugs in the open well of the interceptors. It is possible that the mean escape rate for 1^st^ instar nymphs would have been less had new interceptors, or a fresh layer of talc been applied to the used interceptors. However, our results with used interceptors are more representative of what would be expected under field conditions.

Interceptors were demonstrated to be an effective and simple method for detecting bed bugs [10–11], [41]. They are placed under furniture legs and checked 7–14 d later. Knowing what percentage of the bed bug population will be trapped during a 7 or 14 d period will help determine the population size and the optimum number of interceptors to be installed for detecting bed bugs. Based on this study, an interceptor placed at the bed or sofa caught only 0.11–0.61% of the bed bug population. It should be noted that the interceptors were placed beside the legs of furniture in this study due to the experiment design. In field practice, interceptors are typically placed directly under the furniture legs, unless they do not fit into the interceptors. In occupied apartments, the percent of the bed bug population being captured by an interceptor can vary greatly. We showed in vacant units, adding a CO_2_ source will increase trap catch by 3.8 times. Increasing the number of interceptors or placement period will provide a better estimate about the presence of bed bugs and their distribution.

## Conclusion

M-r-r technique and the use of pitfall-style interceptors are effective methods for studying bed bug movement under field conditions and to estimate bed bug populations. Nymphs and adult bed bugs of both sexes are very mobile and travel extensively throughout apartments. Bed bugs have the ability to disperse from occupied and vacant apartments to neighboring apartments. Bed bugs can survive at least 4.5 months of starvation at field conditions. These findings have important implications on bed bug management and eradication programs. Movement of bed bugs away from predictable locations such as beds and upholstered furniture within apartments may complicate control efforts, making it more difficult to eradicate bed bugs and determine when infestations have been eliminated. The active dispersal of bed bugs between apartments suggests inspecting surrounding units, including apartments across the hallway from known infestations, is necessary.

## Supporting Information

S1 Fig Cumulative movement of marked bed bugs within apartments at 24 h and 14 d after release. Two one bedroom apartments (#2 and 6) were selected to illustrate the movement of the marked bed bugs following release. Marked bed bugs moved from their point of release, to a different room in both apartments within 24 h. Numbers in circles represent the number of marked bed bugs of that color trapped in a particular location.
(TIF)
