# Supplementary figures and images for: Mark-Release-Recapture Reveals Extensive Movement of Bed Bugs (Cimex lectularius L.) within and between Apartments
Source: PLoS One. 2015 Sep 9;10(9):e0136462. doi: 10.1371/journal.pone.0136462 (PMC4564232; doi:10.1371/journal.pone.0136462)

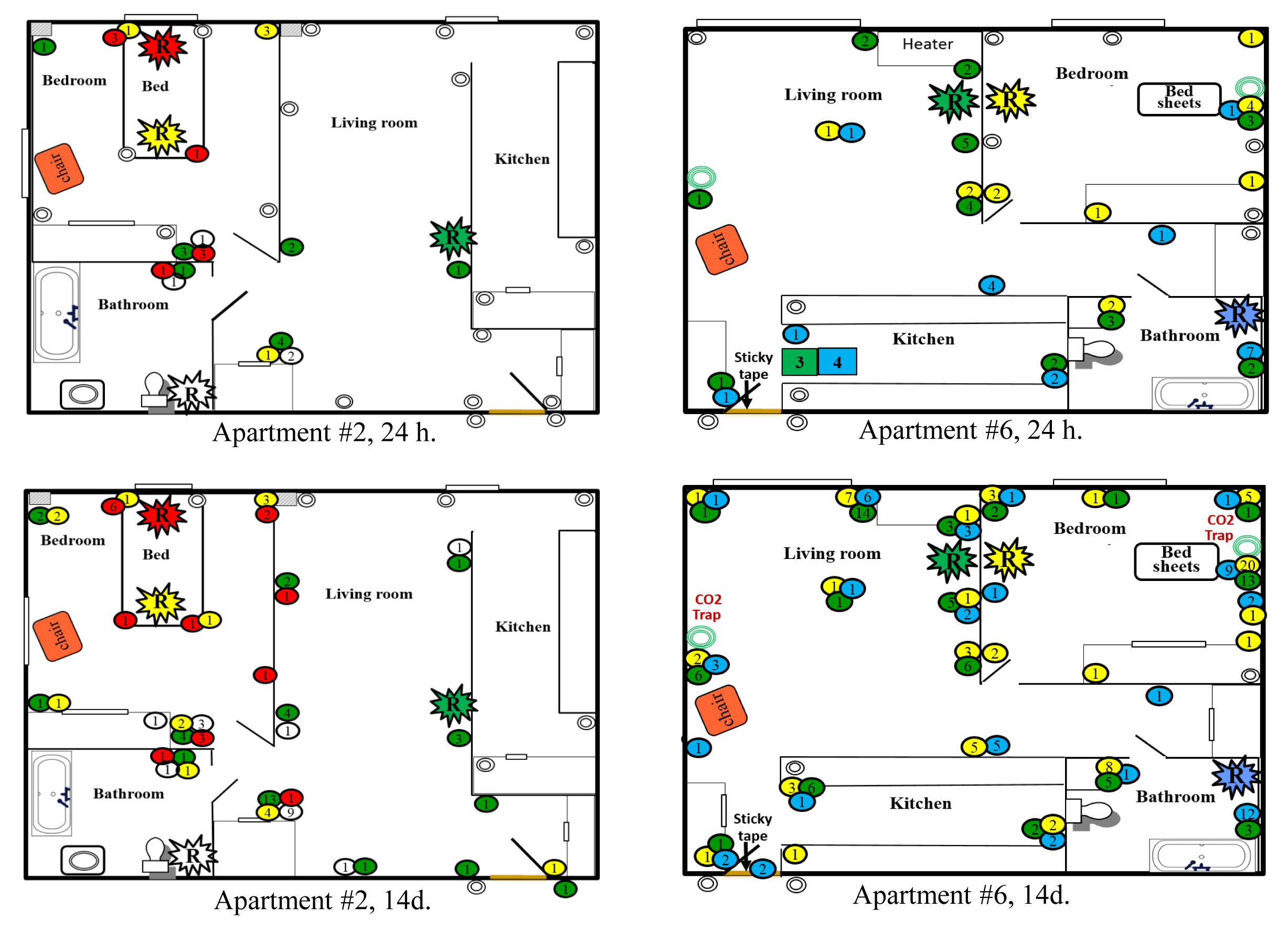

Supplement: S1 Fig — Two one bedroom apartments (#2 and 6) were selected to illustrate the movement of the marked bed bugs following release. Marked bed bugs moved from their point of release, to a different room in both apartments within 24 h. Numbers in circles represent the number of marked bed bugs of that color trapped in a particular location. (TIF) [file pone.0136462.s001.tif]
